# The Multifunctional Role of Poloxamer P338 as a Biofilm Disrupter and Antibiotic Enhancer: A Small Step forward against the Big Trouble of Catheter-Associated *Escherichia coli* Urinary Tract Infections

**DOI:** 10.3390/microorganisms10091757

**Published:** 2022-08-31

**Authors:** Lucia Henrici De Angelis, Mariarita Stirpe, Dario Tomolillo, Gianfranco Donelli, Iolanda Francolini, Claudia Vuotto

**Affiliations:** 1 Microbial Biofilm Laboratory, IRCCS Fondazione Santa Lucia, 00179 Rome, Italy; 2 Department of Science, Roma Tre University, 00154 Rome, Italy; 3 Department of Chemistry, Sapienza University of Rome, 00185 Rome, Italy

**Keywords:** Poloxamer P338, biofilm disruption, catheter-associated urinary tract infections, *E. coli*, antibiotic enhancer

## Abstract

Poloxamer 338 (P338), a nonionic surfactant amphiphilic copolymer, is herein proposed as an anti-biofilm compound for the management of catheter-associated urinary tract infections (CAUTIs). P338’s ability to disrupt *Escherichia coli* biofilms on silicone urinary catheters and to serve as antibiotic enhancer was evaluated for biofilm-producing *E. coli* Ec5FSL and Ec9FSL clinical strains, isolated from urinary catheters. In static conditions, quantitative biofilm formation assay allowed us to determine the active P338 concentration. In dynamic conditions, the BioFlux system, combined with confocal laser scanning microscopy, allowed us to investigate the P338 solution’s ability to detach biofilm, alone or in combination with sub-MIC concentrations of cefoxitin (FOX). The 0.5% P338 solution was able to destroy the structure of *E. coli* biofilms, to reduce the volume and area fraction covered by adherent cells (41.42 ± 4.79% and 56.20 ± 9.22% reduction for the Ec5FSL and Ec9FSL biofilms, respectively), and to potentiate the activity of 1\2 MIC FOX in disaggregating biofilms (19.41 ± 7.41% and 34.66 ± 3.75% reduction in the area fraction covered by biofilm for Ec5FSL and Ec9FSL, respectively) and killing cells (36.85 ± 7.13% and 32.33 ± 4.65% increase in the biofilm area covered by dead Ec5FSL and Ec9FSL cells, respectively).

## 1. Introduction

Microbial cells growing as biofilms on medical device surfaces may show an enhanced antimicrobial tolerance with respect to their free-floating counterparts, due to physical or chemical barriers and increased transmission of resistance markers within biofilms. This tolerance can lead to antibiotic treatment failure, causing difficult-to-treat, medical-device-related infections [1]. Catheter-associated urinary tract infection (CAUTI) is one of the most frequent biofilm-based device-related infections, with both the internal and external surfaces of indwelling catheters being susceptible to biofilm colonization, and is a well-recognized source of increased morbidity and mortality rates, as well as a significant financial burden for the patients, their families, and the healthcare system [2]. In particular, elderly, immunocompromised, and bedridden patients, such as those affected by severe illness or chronic conditions (e.g., cerebrovascular accidents, spinal injury, or neurological diseases such as Alzheimer’s disease, Parkinson’s disease, and multiple sclerosis), often require urinary catheterization, which is a relatively common management option for bladder dysfunction [3,4]. The prolonged use of indwelling urinary catheters leads to a higher risk of acquiring CAUTIs due to the adhesion of multidrug-resistant microorganisms on the catheter’s internal and external surfaces [5,6].

CAUTIs can be caused by various bacterial species, such as *Enterococcus* spp., *Escherichia* spp., *Klebsiella pneumoniae*, *Proteus mirabilis*, *Pseudomonas aeruginosa*, and *Acinetobacter* spp. [7]. Among these bacterial species, the most frequent is *Escherichia coli* [8]. In fact, biofilm-forming *E. coli* strains resistant to the generally used antibiotics—including ceftazidime, tetracycline, meropenem, amikacin, imipenem, fosfomycin, cloxacillin, gentamicin, ciprofloxacin and cefoxitin—are increasingly commonly isolated from the urine of patients (especially hospitalized patients) [7]. Extrapolated from the literature data, a consistent number of multidrug-resistant *E. coli* isolates collected from patients suffering from UTIs are ESBL and AmpC β-lactamase-producing strains [9]. A correlation between ESBL production and biofilm formation ability has been detected [10], and a synergetic effect of ESBLs, AmpC, and biofilms has been revealed recently [11].

Given these premises, the prevention of or therapy against CAUTIs has become a major focus of many hospital infection control programs. During the last decade, great advancements have been made in medical device-related research all over the world, in order to develop new tools and discover non-resistance-inducing molecules able to modulate different steps of biofilm formation or to disperse preformed biofilms [1].

Among others, surfactants are widely used as emulsifiers, detergents, wetting and foaming agents, and dispersants in different areas, from cosmetics to food. Moreover, their use in clinical settings is becoming quite common—mostly in wound care, where a number of surfactant-based cleansers, based for example on poloxamers, show huge potential in enhancing the closure of infected wounds [12]. 

Poloxamers, also called Pluronics, are a unique class of inert amphiphilic poly(ethylene oxide) (PEO) and poly(propylene oxide) (PPO) block copolymers (PEO–PPO–PEO) reported as suitable compounds for biomedical applications such as tissue engineering and drug delivery [13]. However, investigation of poloxamers has increased in recent years due to their cleansing, protein aggregation suppressing, and tissue-repairing abilities, which may also open new scenarios in the field of anti-biofilm strategies, mostly with respect to wound dressing [14,15].

In a previous study [16], we demonstrated that Poloxamer 338 (P338) is able to counteract *E. coli*’s adhesion on silicone catheters, and we developed an in vitro model mimicking phenomena occurring in the urinary system at the micro scale. In this study, P338 was investigated under static and dynamic conditions as a potential anti-biofilm compound to detach *E. coli* biofilms grown on the surface of urinary catheters, alone or in combination with sub-MIC concentrations of cefoxitin (FOX). P338 solution (0.5%) was able to promote *E. coli* biofilm disruption and to act as an antibiotic enhancer, by decreasing the tolerance of *E. coli* biofilms to FOX. This leads us to consider P338 as a promising anti-biofilm agent for CAUTI control.

## 2. Materials and Methods

### 2.1. Materials and Bacterial Strains

P338 (Merck Life Science S.r.l., Milan, Italy) has a molecular weight (Mw) of 14,600 Da, with 50.34 PPO units and 265.45 PEO units [17]. 

The Ec5FSL and Ec9FSL *E. coli* strains, collected from the urine of patients suffering from CAUTIs hospitalized at IRCCS Fondazione Santa Lucia (Rome, Italy), were used. The antibiotic resistance profiles and biofilm phenotypes were described by Stirpe et al. [16]. 

### 2.2. Cefoxitin Disk Diffusion and Minimum Inhibitory Concentration (MIC) Assays

The *E. coli* isolates were screened for susceptibility to FOX, not included in the AST-N202 card, by disk diffusion and broth microdilution methods. Disk diffusion tests were carried out on Mueller–Hinton agar (Oxoid, Rodano, Italy) with 30 μg FOX antibiotic disks (Oxoid). The twofold broth microdilution method was used for determination of FOX MIC (Fisher Scientific, Rodano, Italy). The MIC values for the Ec5FSL and Ec9 FSL isolates were determined as described by Wiegad et al. [18], and considered as the lowest concentration of FOX that prevented visible growth of *E. coli* isolates in 96-well polystyrene round-bottomed microwell plates. 

FOX resistance (R) was defined as a zone diameter < 19 mm obtained by the standard disk diffusion method using a 30 μg disk, or an MIC > 8 mg/L (corresponding to the ECOFF) determined by the broth microdilution method [19].

### 2.3. Phenotypic AmpC Confirmation Testing

To phenotypically reveal AmpC β-lactamase production, the strains were inoculated on Mueller–Hinton (MH) agar using a 0.5 McFarland bacterial suspension, and incubated at 37 °C overnight, using FOX (30 μg) and FOX/phenylboronic acid (30/400 μg) disks. A ≥5 mm increase in zone diameter in the presence of phenylboronic acid against FOX alone was considered positive for the production of AmpC β-lactamase [20].

### 2.4. Biofilm Dispersion Assay of E. coli with P338 under Static Conditions

The efficacy of P338 solution at different concentrations in dispersing *E. coli* biofilms grown under static conditions was determined by using 96-well flat-bottomed plastic tissue culture plates. Ec5FSL and Ec9FSL cultures were diluted in LB broth to obtain a 0.1 OD_600_ bacterial suspension; 20 μL dilutions were used to inoculate wells filled with 180 μL of LB broth supplemented with 1% glucose (*w*/*v*). Sterile broth was used as a negative control. Plates were incubated for 20 h at 37 °C. After that, each well was washed three times with PBS, and then P338 solutions ranging from 5 mg/mL (0.5%) to 1.25 mg/mL (0.125%), obtained by twofold dilutions in PBS, were added to each well and incubated for 6 h. In the positive control, PBS alone was added. Then, the wells were emptied and washed three times with PBS. Finally, quantification of biomass was defined by performing crystal violet (CV) staining as previously described [21]. 

### 2.5. Biofilm Dispersion Assay of E. coli with P338 under Dynamic Conditions

Microfluidic channels of the BioFlux System 200 (Fluxion Biosciences, Inc., Alameda, CA, USA) were inoculated with the Ec5FSL and Ec9FSL strains (OD_600_ = 0.25). One hour later, non-adherent bacteria were washed out of the channel through the waste well, and the experiment was run for 18 h, supplementing LB broth supplemented with 1% glucose (*w*/*v*) at 0.5 dyne/cm^2^ [16]. After achieving a mature biofilm, it was treated as suggested by Diaz et al. [22] (modified for our purposes). A 0.5% P338 solution diluted in PBS was instilled within the microfluidic channel at 0.5 dyne/cm^2^ for 6 h to assess its ability to detach or disaggregate biofilms. As a negative control, PBS alone was introduced to the system once a mature biofilm was obtained. A time-lapse recording was taken to monitor the biofilm development, by taking a sequence of frames at set intervals (2 min to monitor the biofilm formation and 1 min to follow the effects of P338 on preformed biofilms) from the beginning to the end of the dynamic experiments.

### 2.6. Minimum Biofilm Eradication Concentration (MBEC) Assay

A modified MBEC assay was performed in 96-well flat-bottomed microplate format. Ec5FSL and Ec9FSL cultures were diluted with LB broth to obtain a 0.1 OD_600_. Then, 20 μL samples of these dilutions were used to inoculate wells filled with 180 μL of LB broth supplemented with 1% glucose (*w*/*v*). Biofilms were allowed to form for 20 h at 37 °C. After that, each well was washed three times with PBS, and then FOX solutions in MH broth—ranging from 4× MIC to ¼ MIC concentrations—were added to each well for 18 h. Then, the FOX solutions were removed, and the wells were washed three times with PBS. The MBEC was finally determined by performing CV staining as described in [23] (modified for our purposes).

### 2.7. Anti-Biofilm Efficacy Test under Dynamic Conditions against E. coli with A Combination of P338 and Cefoxitin 

Microfluidic channels were inoculated with the Ec5FSL and Ec9FSL strains (OD_600_ = 0.25), and biofilms were obtained as described in Section 2.5. After achieving a mature biofilm, a mixture of 0.5% P338 solution and FOX at ½ MIC was instilled within the microfluidic channel at 0.5 dyne/cm^2^ for 3 h to assess the ability of P338, so as to improve the efficacy of FOX against preformed *E. coli* biofilms. As a negative control, PBS alone was introduced to the system once a mature biofilm was obtained. A time-lapse recording was taken to monitor the biofilm development, by taking a sequence of frames at set intervals (every 2 min to monitor the biofilm formation and every 1 min to follow the effects of 0.5% P338/½ MIC FOX solution against preformed biofilms) from the beginning to the end of the dynamic experiments, as described in [23] (modified for our purposes).

### 2.8. Confocal Laser Scanning Electron Microscopy (CLSM)

*E. coli* biofilms grown within microfluidic channels were stained with the LIVE/DEAD BacLight Bacterial Viability Kit (L-7007, Thermo Fisher Scientific, Waltham, MA, USA). This kit allowed us to discriminate between live and dead cells, with intact cells staining fluorescent green (SYTO^®^ 9), whereas damaged bacteria stained fluorescent red (propidium iodide). In detail, a 0.85% NaCl solution with 6 μM SYTO 9 and 30 μM propidium iodide was instilled within the microfluidic channel for 30 min at 0.4 dyne/cm^2^. After that, a 0.85% NaCl solution was used to wash the channel for 20 min at 0.2 dyne/cm^2^ [24] (modified for our purposes). The entire length of the channel designed for BioFlux visualization, divided into different sections with an area of 0.234 mm^2^, was examined by CLSM (Nikon mod. C1si) at 20X magnification (WD 3.1). Recorded datasets obtained by CLSM were used for both visualization and semi-quantitative analysis by counting pixels (2D) after thresholding the raw dataset.

### 2.9. Statistical Analysis

The OD_570_ values of static biofilms treated with different concentrations of P338, along with recorded datasets obtained by CLSM, were used for statistical analysis. 

Statistical analyses of data were carried out using GraphPad Prism software (version 6.01; GraphPad Software, San Diego, CA 92108, USA). Data on static biofilms, derived from three independent experiments, are presented as the mean OD_570_ values ± standard deviation. Data obtained through CLSM are presented as the mean surface covered by either biofilm or dead cells in the biofilm-covered area ± standard deviation calculated after the removal of outliers (ROUT method, Q = 1%). At least four different biofilm areas were considered for each experimental condition. Statistical analyses of differences between means and their standard errors were performed using Welch’s unequal variances *t*-test, considering unpaired datasets. A *p*-value lower than 0.05 was considered to be statistically significant.

### 2.10. Ethical Statement

The Biobank of the Fondazione Santa Lucia, Rome, Italy, has been ethically approved as a research bank by the Ethical and Protocol Review Committee, with the protocol identification number “CE/PROG.796”.

## 3. Results

The use of poloxamers as anti-biofilm compounds for indwelling medical devices is still poorly understood. The aim of this study was to explore, using a dynamic in vitro catheter model, the efficacy of P338 as an anti-biofilm/disrupting agent against preformed biofilms of two phenotypically different *E. coli* strains causing CAUTIs. 

### 3.1. Cefoxitin Resistance Profile

The two *E. coli* isolates were previously characterized for antibiotic resistance profiles and biofilm formation abilities in [16]. The Ec5FSL isolate was classified as susceptible (S) and strongly adherent, whilst the Ec9FSL isolate was considered to be multidrug-resistant (MDR), with an ESBL-producing drug resistance profile, and moderately adherent.

Resistance to FOX was also tested. The disk diffusion method classified Ec9FSL as also being resistant to FOX, and the broth microdilution test defined the corresponding MIC = 8 mg/L (Table 1).

A FOX zone diameter < 19 mm, combined with phenotypic resistance to ceftazidime and/or cefotaxime, as in the case of the Ec9FSL isolate, may be used as phenotypic criteria for the investigation of AmpC β-lactamase production, although this strategy will not detect ACC-1—a plasmid-mediated AmpC that does not hydrolyze cefoxitin [25]. More precise phenotypic AmpC confirmation tests are generally based on inhibition of AmpC by either cloxacillin or boronic acid derivatives. A combined disk method using the combination of cefoxitin (30 μg) and cefoxitin/phenylboronic acid (30/400 μg) was used as a screening marker for AmpC production in the FOX-resistant Ec9FSL isolate. No increase in zone diameter was detected, thus characterizing the Ec9FSL as an ESBL+/AmpC− strain.

### 3.2. E. coli Biofilm Dispersion after P338 Treatment under Static Conditions

P338 was evaluated for its ability to disperse preformed biofilms of susceptible and MDR *E. coli* isolates.

**Figure 1:**
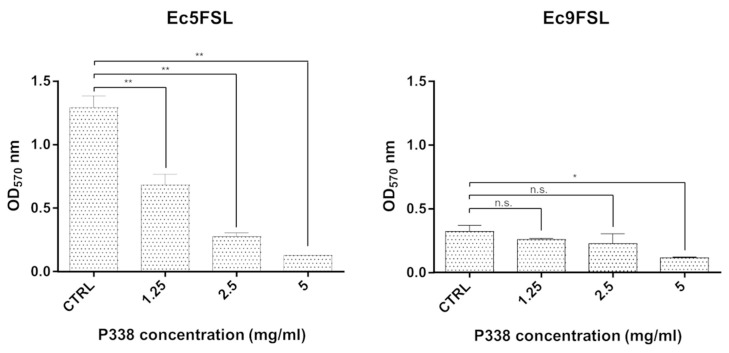
**Different P338 concentrations reduce the biofilm biomass of *E. coli* isolates in static conditions:** OD_570_ values of CV-stained Ec5FSL and Ec9FSL untreated biofilms (CTRL) treated for 6 h with different P338 concentrations. The results are shown as the mean ± standard deviation of the values obtained from three independent experiments under each condition; * *p*-value < 0.05 (confidence interval: 95%), ** *p*-value < 0.01 (confidence interval: 99%), n.s. = “not significant” by Welch’s unequal variances *t*-test.

Primarily, the experiments were carried out under static conditions in 96-well flat-bottomed plastic tissue culture plates (Figure 1) to define a more reasonable concentration of P338 solution to be used under dynamic conditions to effectively disrupt preformed *E. coli* biofilms.

After 6 h of treatment with different concentrations of P338 solution, a dose-dependent efficacy was detected within the range from 5 mg/mL to 1.25 mg/mL. The minimum P338 concentration showing a statistically significant biofilm reduction (*p* < 0.05) for both isolates was 5 mg/mL (0.5%) (Figure 1), with a 90.10 ± 4.13% and 63.97 ± 8.59% reduction in biofilm biomass for the Ec5FSL and Ec9FSL isolates, respectively.

### 3.3. E. coli Biofilm Dispersion after P338 Treatment under Dynamic Conditions

P338 at a 5 mg/mL (0.5%) concentration was chosen to verify its biofilm disruption ability under dynamic conditions. The dynamic system in this study mimics silicone urinary catheters occluded by *E. coli* biofilms. 

**Figure 2:**
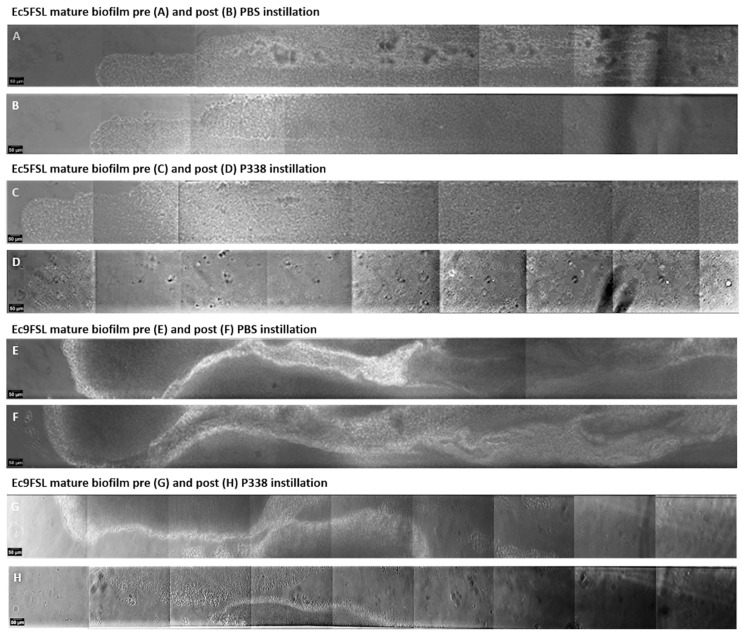
**P338 disrupts biofilm biomass in a urinary catheter in vitro model:** Reconstruction of portions of viewing windows of the microfluidic channels containing the Ec5FSL and Ec9FSL biofilms immediately before (**A**,**C**,**E**,**G**) and after 6 h instillation of 1× PBS (**B**,**F**) or 0.5% P338 solution (**D**,**H**). Acquisition was performed with a 20×, 3.1 WD objective.

To this end, mature Ec5FSL and Ec9FSL biofilms, formed within BioFlux microfluidic channels made of polydimethylsiloxane (PDMS), were treated with 0.5% P338 solution to assess its ability to detach/disrupt *E. coli* biofilms. As a negative control, 1× PBS was introduced to the system once a mature biofilm was obtained (Figure 2). 

As revealed from the reconstruction of the portion of the viewing windows of microfluidic channels containing the Ec5FSL and Ec9FSL biofilms after 6 h of treatment, 0.5% P338 solution was able to detach a greater portion of the Ec5FSL (Figure 2C,D) and Ec9FSL biofilms (Figure 2G,H) compared to the control (1× PBS) (Figure 2A,B,E,F, respectively). As for Ec5FSL, only monostratified cells remained attached to the channel after the instillation of P338 (Figure 2C,D), with the defects in the silicone tube’s surface clearly visible, while for Ec9FSL, 0.5% P338 solution was able to disrupt entire macrocolonies formed within the microfluidic channel.

**Figure 3:**
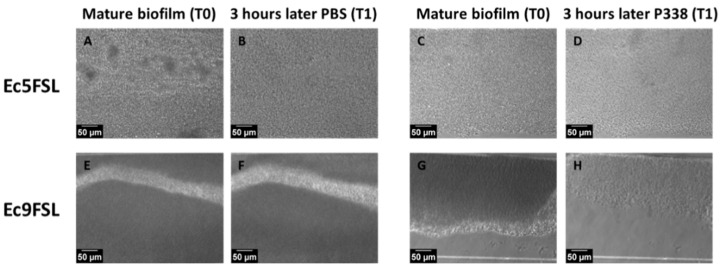
**Early anti-biofilm activity of P338 detected by frames extrapolated from BioFlux time-lapse recording:** BioFlux images of 15 h old Ec5FSL (**A**,**C**) and Ec9FSL (**E**,**G**) biofilms, and 3 h after the instillation of PBS ((**B**,**F**), respectively) and 0.5% P338 solution ((**D**,**H**), respectively). Acquisition was performed with a 20×, 3.1 WD objective.

However, by monitoring the AVI files obtained from the P338 solution treatment over time, and by observing each frame constituting them, we noticed that a significant reduction in the Ec5FSL and Ec9FSL biofilms could already be detected after 3 h (Figure 3). 

The micrographs shown in Figure 3 were obtained by extrapolating the frames obtained during 6 h of treatment with PBS and P338 alone against mature biofilm (Section 2.5). Substantial differences were found when 0.5% P338 solution was instilled against mature biofilms (Figure 3D,H), with respect to the control (Figure 3B,F), in terms of the biofilms’ three-dimensional structure and thickness (grayscales).

**Figure 4:**
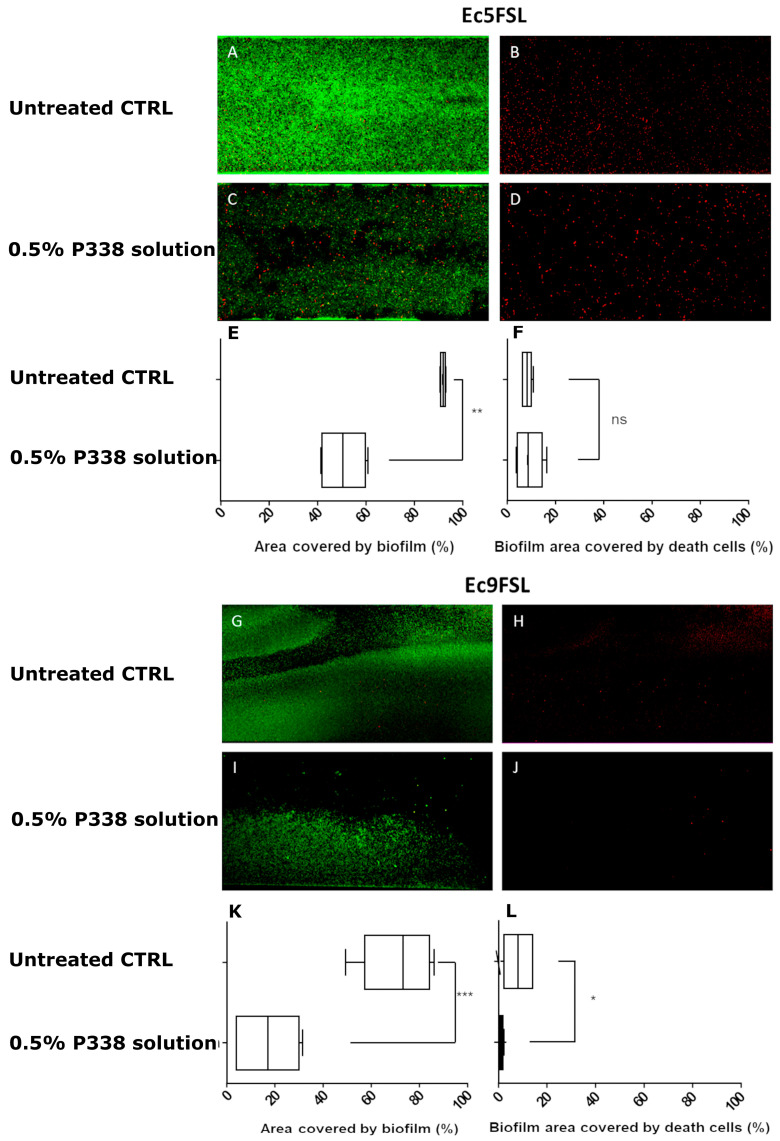
**P338 exhibits anti-biofilm properties without impairing cell viability:** Representative CLSM images of Ec5FSL (**A**,**B**) and Ec9FSL (**G**,**H**) biofilm controls, and after 3 h of 0.5% P338 solution instillation against Ec5FSL (**C**,**D**) and Ec9FSL (**I**,**J**) mature biofilms, obtained using the LIVE/DEAD BacLight Bacterial Viability Kit. The area fraction covered by biofilm (i.e., area of total biomass constituted by live and dead cells) and the biofilm area covered by dead cells (i.e., area of dead cells within the biofilm) for the Ec5FSL (**E**,**F**) and Ec9FSL (**K**,**L**) isolates are reported as percentages obtained as the mean ± s.d. of at least 4 images. Statistical significance of the percentages of each treatment and with respect to the untreated controls are represented as follows: * *p*-value < 0.05 (confidence interval: 95%); ** *p*-value < 0.01 (confidence interval: 99%); *** *p*-value < 0.001 (confidence interval: 99.9%). ns = not significant.

The area fraction covered by biofilm and the effects in terms of percentage of dead cells within the biofilm after P338 treatment were determined by semi-quantitative analysis of several CLSM images, obtained using the LIVE/DEAD BacLight Bacterial Viability Kit, collected after 3 h treatments (Figure 4).

Taking into consideration the CLSM images and the percentages reported in Figure 4, after 3 h of treatment, the 0.5% P338 solution significantly reduced the Ec5FSL (41.42 ± 4.79%; *p*-value 0.003) (Figure 4C,E) and Ec9FSL (56.20 ± 9.22%; *p*-value: 0.0005) (Figure 4I,K) biofilms’ biomass with respect to the controls (Figure 4A,G, respectively), not notably influencing the percentage of dead cells within the biofilm (Figure 4D vs. Figure 4B,F, and Figure 4J vs. Figure 4H,L respectively). 

### 3.4. Cefoxitin Minimum Biofilm Eradication Concentration (MBEC) against E. coli

**Figure 5:**
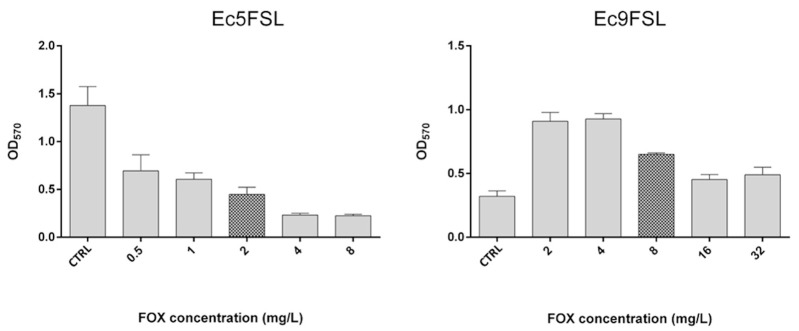
**MBEC of Cefoxitin against *E. coli* isolates:** MBEC results of Ec5FSL and Ec9FSL biofilms treated with different FOX solutions. The dotted bars indicate the FOX MIC concentrations for Ec5FSL and Ec9FSL isolates.

FOX was tested against both isolates grown as biofilms, in order to evaluate some variability in the susceptibility of the *E. coli* isolates’ biofilms to FOX concentrations ranging from 4× MIC to ¼ MIC (Figure 5).

At the tested concentrations, Ec9FSL showed a greater survival in biofilms compared to Ec5FSL and, even more interestingly, an increase in biofilm biomass with respect to the control was observed, as previously seen when sub-MIC concentrations were used against biofilms [26].

On the other hand, the Sc5FSL biofilm biomass was reduced by FOX, in a concentration-dependent manner (Figure 5). Nevertheless, when comparing the MIC and MBEC results, the susceptibility to FOX of strains growing as biofilms, under static conditions, was lower than that of planktonic microorganisms. The biofilm biomass was 0.45 (at 2 mg/L) and 0.65 (at 8 mg/L) OD_570_ for Ec5FSL and Ec9FSL, respectively. 

### 3.5. Anti-Biofilm Efficacy of a Combination of P338 and Cefoxitin against E. coli under Dynamic Conditions

**Figure 6:**
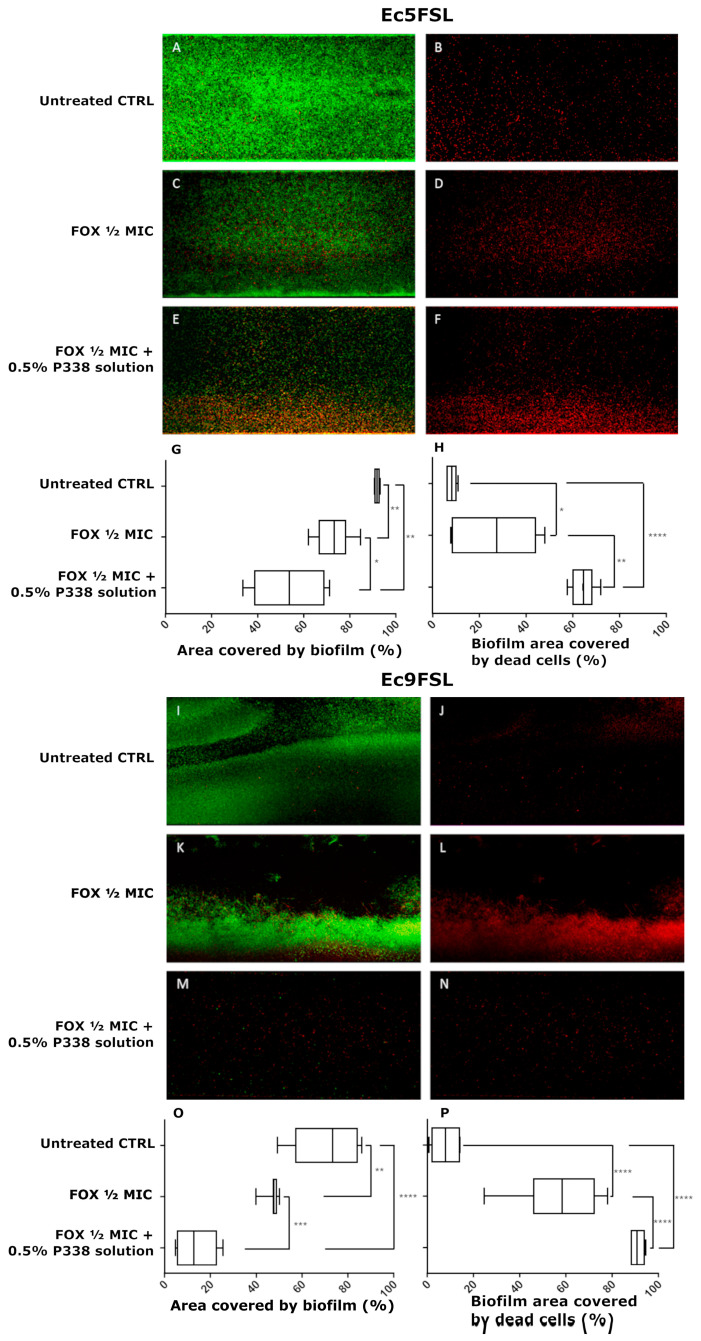
**P338 synergizes with Cefoxitin to impair biofilm cell viability:** Representative CLSM images, obtained by using the LIVE/DEAD BacLight Bacterial Viability Kit, of Ec5FSL (**A**,**B**) and Ec9FSL (**I**,**J**) biofilm controls, after 3 h of ½ MIC FOX solution instillation against mature Ec5FSL (**C**,**D**) and Ec9FSL (**K**,**L**) biofilms, and after 3 h of ½ MIC FOX/0.5% P338 solution instillation against mature Ec5FSL (**E**,**F**) and Ec9FSL (**M**,**N**) biofilms. Area fractions covered by biofilm (i.e., area of total biomass constituted by live and dead cells) and biofilm areas covered by dead cells (i.e., area of dead cells within the biofilm) for Ec5FSL (**G**,**H**) and Ec9FSL (**O**,**P**) isolates are graphed as percentages obtained from the mean ± s.d. of at least 4 images. Statistical significance of the percentages of each treatment and with respect to the untreated controls are represented as follows: * *p*-value < 0.05 (confidence interval: 95%); ** *p*-value < 0.01 (confidence interval: 99%); *** *p*-value < 0.001 (confidence interval: 99.9%); **** *p*-value < 0.0001 (confidence interval: 99.99%).

The efficacy of 0.5% P338 solution in enhancing the antibiotic activity against biofilms and/or reducing the antibiotic tolerance of *E. coli* grown as biofilms was evaluated by instilling a mixture of FOX and P338 solution within microfluidic channels. Based on the results obtained by MBEC assay, FOX at ½ MIC was chosen in order to better detect any differences in FOX’s efficacy against *E. coli* biofilms when a mixed 0.5% P338/½ MIC FOX solution was added, compared to the addition of FOX at ½ MIC alone (Figure 6).

As can be inferred from the CLSM images and the graphs, the 0.5% P338 solution was able to significantly increase the efficacy of FOX against biofilm-grown cells of both Ec5FSL and Ec9FSL isolates, in terms of both the area covered by biofilm (Figure 6G,O) and the biofilm area covered by dead cells (Figure 6H,P). More specifically, the ½ MIC FOX/0.5% P338 solution mixture was able to reduce the Ec5FSL biofilm biomass by 38.08 ± 6.74% (*p*-value 0.0023) with respect to the untreated control, as opposed to 18.66 ± 3.16% (*p*-value 0.0017) when ½ MIC FOX solution alone was used. An increase in the anti-biofilm efficacy of 19.41 ± 7.41 was detected when 0.5% P338 was added to ½ MIC FOX (Figure 6G). More interestingly, a 56.18 ± 2.20% (*p*-value < 0.0001) increase in cell death was found when ½ MIC FOX/0.5% P338 solution was added to biofilm compared to the untreated control, whilst ½ MIC FOX solution alone only caused an increase in cell death of 19.33 ± 6.90% (*p*-value 0.0366). This means that the ½ MIC FOX/0.5% P338 solution produced a 36.85 ± 7.13% increase in cell death compared to ½ MIC FOX solution alone (Figure 6H).

As for Ec9FSL, the area covered by biofilm treated with the ½ MIC FOX/0.5% P338 solution mixture resulted in a decrease of 60.46 ± 7.13% (*p*-value < 0.0001) with respect to the control, as opposed to 25.80 ± 6.20% (*p*-value 0.0081) when ½ MIC FOX solution alone was used. A variation of 34.66 ± 3.75% was found between the ½ MIC FOX/0.5% P338 solution mixture and ½ MIC FOX solution alone (Figure 6O). With regard to the percentage of dead cells, an increase of 82.86 ± 2.69 (*p*-value < 0.0001) was observed when ½ MIC FOX/0.5% P338 solution was added to the Ec9FSL biofilm with respect to the control, and an increase of 32.33 ± 4.65% (*p*-value < 0.0001) when compared to ½ MIC FOX solution alone (50.53 ± 5.14% increase in cell death vs. CTRL) (Figure 6P).

## 4. Discussion

Medical implants frequently have to be removed because of bacterial infection [27]. The biomaterial is colonized, and bacteria develop an environment that protects them from host defenses and antibiotics [28].

The identification of effective, non-biocidal compounds capable of counteracting the formation of biofilms on medical devices, causing neither toxicity nor the development of resistance, is increasingly necessary.

Poloxamers are a family of over 50 different amphiphilic nonionic polyoxyethylene and polyoxypropylene block copolymers, consisting of a central hydrophobic PPO block and two lateral hydrophilic PEO blocks, widely used in in cosmetics at concentrations ranging from 0.005% to 20%, as surfactants, or as solubilizing or cleansing agents. Overall, they are generally nontoxic to animals (LD_50_ from 5 to ca. 35 g/Kg), and have a rapid clearance if introduced into the body [29]. Several medical applications for poloxamers have been investigated recently [13].

Their surfactant activity, safety profile, and biocompatibility have prompted researchers to investigate the role that poloxamers play in biofilm management, matrix metalloproteinase modulation, inflammation reduction, and enhancement of cellular proliferation, behavior, and viability.

A far as we know, the activity of poloxamers on preformed biofilms has been demonstrated in wound infections, with Yang et al. proving the efficacy of a daily application of a poloxamer in reducing the levels of mature biofilm grown on porcine skin explants [30]. In the field of infections related to implantable devices, few research groups have proposed the use of poloxamers as candidate compounds for catheter coatings [16,31], but nobody has ever suggested the application of poloxamers as anti-biofilm compounds to disaggregate biofilms already formed on urinary catheters. 

Regarding the exploited methodologies, a number of research groups have used the BioFlux system to evaluate the anti-biofilm effects of different antibiotics [32,33]. Some papers have been published on the use of the BioFlux system to determine the anti-biofilm ability of natural [34] and surfactant [35] compounds; some others have studied the synergistic effects of natural/synthetic molecules [36,37]. In contrast, the evaluation of the anti-biofilm efficacy of a surfactant–antibiotic combination in a BioFlux system mimicking a urinary catheter is quite original.

In this study, we selected Poloxamer P338, aware of its already-demonstrated antifouling properties under conditions resembling those of urinary catheterization [16]. We can hypothesize that, since nonionic surfactants are commonly used in biology to aid in the solubilization of proteins and lipids [38], nonionic surfactants may solubilize the extracellular biomolecules of the biofilm matrix, thus weakening the structure and interfering with the aggregated bacteria in biofilms. A similar biofilm-disaggregating action towards *Staphylococcus aureus* and *Pseudomonas aeruginosa*, attributed to a surfactant-induced dissolution of the biofilm matrix, was also demonstrated for the surfactant Poloxamer P188 embedded in a wound dressing [39].

We showed that a 0.5% P388 solution was able to significantly reduce the biofilm biomass of clinical isolates of *E. coli* collected from urinary catheters. Some differences in biofilm reduction rates were observed between static and dynamic experiments for both strains, with the former providing higher efficacy rates. These results, attributed mainly to the different materials of the surfaces as well as to the number of variables introduced with a dynamic system, grant us understanding of how biologically relevant it is to choose the best in vitro experimental system that mimics the conditions in vivo, in order to obtain the most reliable results.

P338 was also demonstrated to serve as an antibiotic adjuvant for biofilm-producing *E. coli* strains, enhancing the activity of FOX in disaggregating biofilms and killing cells of Ec5FSL and Ec9FSL isolates. FOX was selected for its good in vitro activity and stability because of a 7-α-methoxy group that inhibits the action of ESBLs [40]. Given its pharmacological properties, FOX appears to be a good carbapenem-sparing agent in the management of urinary tract infections caused by ESBL-producing *E. coli* [41,42,43] because, unlike oxyimino-cephalosporins, it is stable against ESBLs, and has been repurposed to treat ESBL-producing *E. coli* UTIs [44,45]. From our perspective, the Ec5FSL isolate was susceptible to FOX, while Ec9FSL turned out to be resistant to FOX on the basis of the disk diffusion method, even if phenotypic AmpC confirmation tests did not demonstrate AmpC production. In addition, regardless of the intrinsic resistance of bacterial strains, an increased tolerance to FOX with respect to the planktonic mode of growth was revealed for Ec5FSL and Ec9FSL cells growing as biofilms. This is consistent with data from the literature [46,47]. These results suggest that under in vivo conditions, FOX, if used alone, although at MIC, might kill free-floating bacteria, but might also fail in the eradication of bacterial cells grown as biofilms.

In order to better underline the improvement provided by P338 in FOX’s effectiveness against *E. coli* biofilms, we decided to use ½ MIC FOX for each strain. By using the microfluidic system, associated with CLSM analysis, 0.5% P338/½ MIC FOX solution, instilled within the silicone channels in which the biofilms were grown, was able to significantly reduce the biofilm biomass and increase cell death for both strains compared to ½ MIC FOX solution alone. Coherently, Yang et al. [30] demonstrated the ability of P188 to sensitize *P. aeruginosa* biofilms to gentamycin intervention in a porcine skin explant model.

By analyzing the data obtained as a whole, the MDR Ec9FSL isolate suffered most from the effects of the different treatments, stressing the huge weight that the biofilm formation ability has on the success of an antibiotic treatment, despite the inherent resistance of a strain. The compactness of the Ec5FSL biofilm, visible through the images acquired with BioFlux and by CLSM, as well as the scarcity of large channels within it, makes it more difficult for the compounds to penetrate, even if the results in any case indicate a significant variation. Despite the astonishing efficacy of the combined FOX/P338 solution in terms of dead cells against the MDR Ec9FSL isolate foreshadowing that P338 could be a very promising alternative to counteract CAUTIs caused by multidrug-resistant strains of *E. coli*, concrete evidence can only be obtained by increasing the antibiotic panel to test and the number of strains, so as to improve the statistical power of our study. We could extend these kinds of experiments to other bacterial species among those mainly related to CAUTI, which would certainly provide greater solidity and robustness to our results and our conclusions, in terms of the non-species-specific efficacy of the P338 compound under examination.

## 5. Conclusions

*E. coli* biofilms appeared to be more susceptible to FOX activity when P338 was instilled at the same time, with the sensitization of otherwise insensitive biofilms presumably being related to the emulsification of the biofilm’s extracellular polymeric substances (EPS) matrix itself via the surfactant action. Overall, our data suggest that the combination of P338 with antibiotics could be a promising approach for therapeutic interventions in case of CAUTIs.

## Figures and Tables

**Table 1:** FOX susceptibility tests against the Ec5FSL and Ec9FSL isolates.

Strains	FOX Zone Diameter ^a^ (mm)	FOX MIC ^c^ (mg/L)	Interpretation
Ec5FSL	23 ^b^	2	S
Ec9FSL	16 ^b^	8	R

^a^ Agar diffusion method. ^b^ Diameter breakpoint for FOX (disk content 30 mg): S ≥ 19 mm; R < 19 mm. ^c^ Broth microdilution MIC determination.

## Data Availability

Not applicable.
